# Maternal carryover drives post-weaning survival of juveniles in a long-lived mammal

**DOI:** 10.1007/s00442-026-05953-y

**Published:** 2026-08-07

**Authors:** Tayler N. LaSharr, Rhiannon P. Jakopak, Taylor O. Wagstaff, Rebekah T. Rafferty, Samantha P. H. Dwinnell, Jill Randall, Cheyenne Stewart, Rusty C. Kaiser, Mark Thonhoff, Brandon Scurlock, Troy Fieseler, Gary L. Fralick, Kevin L. Monteith

**Affiliations:** 1 Haub School of Environment and Natural Resources, University of Wyoming, Bim Kendall House, 804 E Fremont St, Laramie, WY 82072 USA; 2 Department of Wildland Resources, Utah State University, Logan, USA; 3 Department of Arctic Biology, University Centre in Svalbard, P.O. Box 156, Longyearbyen, NO-9171 Norway; 4 The Department of Ecology and Natural Resource Management, Norwegian University of Life Sciences, P.O. Box 5003, Ås, NO-1432 Norway; 5 Wyoming Game and Fish Department, Pinedale Regional Office, 432 Mill St, Pinedale, WY 82941 USA; 6 Wyoming Game and Fish Department, Jackson Regional Office, 420 North Cache, Jackson, WY 83001 USA; 7 U.S. Forest Service, Big Piney Ranger District, 10418 South US Highway 189, Big Piney, WY 83113 USA; 8 Bureau of Land Management, Pinedale Field Office, 1625 West Pine St, Pinedale, WY 82941 USA

**Keywords:** Decision making, Fitness, Migration, Mule deer, Nutrition

## Abstract

The decisions and conditions of animals often carry over to influence fitness in subsequent seasons or years. The condition, choices, and behaviors of parents may have a huge influence on offspring survival, not only when they are nutritionally dependent (i.e., pre-weaning or pre-fledgling), but also after they have reached independence but before adulthood (i.e., post-weaning or post-fledgling). Using a long-lived, iteroparous mammal, we evaluated the hypothesis that carryover effects of a mother’s condition (i.e., age and nutritional condition) and her behavior and decisions during the first few months of her offspring’s life will influence survival of juvenile deer after they reach nutritional independence—during their first migration and their first winter. Age of a mother and mass of her neonates at birth influenced if her offspring survived their first winter; mothers who were young or had high body fat produced offspring that were more likely to survive winter. Moreover, mothers could improve overwinter survival of their offspring by delaying the initiation of their autumn migration. Internal characteristics (i.e., body fat and age) and decisions of parents during their offspring’s first year of life carried over to influence survival of those juveniles after they become nutritionally independent. Juvenile animals still carry the costs and face the consequences of their mother’s decisions well into the period of their independence. The influence of parental characteristics on offspring survival once independent demonstrates the importance of considering how environmental and behavioral characteristics influence survival across generations.

**Supplementary Information:**

The online version contains supplementary material available at https://doi.org/10.1007/s00442-026-05953-y.

## Introduction

In seasonal environments, identifying the choices, behaviors, or physiological responses of an animal in one season can be instrumental to understanding its biological or behavioral responses in following seasons (Harrison et al. 2011; Monteith et al. 2013; O’Connor and Cooke 2015; Sawyer et al. 2019a). Extensive evidence has demonstrated the importance of considering the effect of previous experiences on the fitness of long-lived animals; the conditions animals are exposed to and the decisions they make throughout the year—from their choice of seasonal ranges to migratory routes—can influence their net energy intake and thus, their ability to regulate reproduction and survival across time (Imlay et al. 2021; Pancerasa et al. 2022; Sawyer et al. 2019a). In seasonal environments, wild animals make decisions and allocate their limited time and energy to reproduction while ideally still securing their own survival (Stearns 1992). Yet, if an animal makes a poor choice in one season, it may come at a substantial fitness cost later in life. Adults often can compensate for a poor decision or an unexpected energetic cost through shifts in behavior or energy allocation without facing long-term fitness consequences (Verzuh et al. 2021). The decisions or conditions of adults, however, can affect more than just themselves; a poor choice by a parent may have little influence on their own lives, but may come at an extreme fitness cost if it affects the immediate or long-term survival of their young.

Juveniles are the most sensitive demographic of populations and can be more responsive to environmental stressors than adults; survival to first reproduction is critical for parental fitness and population persistence (Gaillard et al. 2000; Gaillard and Yoccoz 2003; Stearns 1992). Across taxa, there is extensive evidence demonstrating the role of parental conditions on the success of young animals in their first few months of life (Carroll and Brown 1977; Hurley et al. 2020; Kautz et al. 2019; Muthersbaugh et al. 2024), but it is less clear how the choices or condition of a parent influence the survival of their offspring once those offspring have become nutritionally independent (i.e., post-weaning or post fledging). Recent evidence, however, has demonstrated the role that parental condition can play on survival to adulthood—for example, maternal condition underpinned survival of mule deer (*Odocoileus hemionus*) in a temperate system (Lamb et al. 2023), and maternal age can shape fitness traits of offspring well past their first year of life in wild sheep (*Ovis aries;* Ravindran et al. 2025). Carryover effects may have huge implications for parental fitness if independent juveniles are faced with an elevated risk of mortality stemming from the decisions their parents made when those offspring were still nutritionally dependent (i.e., pre-weaning). For long-lived species, animals spend the first season of their lives growing rapidly and building stores of capital necessary to survive their first winter when resources become scarce, and weather becomes inclement (Anderson and Scharf 2014; Heffelfinger and Heffelfinger 2023; Jones et al. 2017). Consequently, the choices made by parents in the summer, while offspring are still developing, may profoundly affect survival of juveniles as they begin to independently navigate the world. Post-weaning survival of juveniles has been well documented in ungulates (Gaillard et al. 2000; Hurley et al. 2020; Lamb et al. 2023, Fouda et al. 2024), but the mechanisms linking parental behaviors or choices to survival of nutritionally independent juveniles are less clear.

For parents to receive fitness benefits from their young, their offspring must survive to first reproduction (Stearns 1992). For many long-lived animals in seasonal environments, young animals must successfully survive both their first migration and winter season to reach adulthood and have the opportunity to reproduce. The risks associated with these two critical periods, however, may be vastly different. For many migratory species, juvenile animals undergo their first migration with their parents (Byholm et al. 2022), and while they do often have older animals to follow, the route itself is completely novel to the young animals. The decisions that parents make, from their choice of route to timing of migration, may expose animals to increased risk of predation, accidents, or sub-optimal forage. If animals succeed in completing their first migration, they then must navigate a winter landscape with limited resources and harsh weather. Winter can pose heightened risks of mortality as populations undergo periods of resource scarcity and potentially severe conditions that last 4–5 months wherein survival depends heavily on stored capital built up during summer when resources are abundant (Lamb et al. 2023; LaSharr et al. 2023c; Remeš and Matysioková 2016). Juvenile animals may face the consequences of decisions made by their parents, even once nutritionally independent because the growth and capital obtained during the summer that affects overwinter survival was heavily dependent on their parent(s). Although difficult to document and often overlooked, identifying the influence of parental decisions in one season on survival of offspring in subsequent seasons, is central to understanding both fitness and population dynamics.

Using nutritional, spatial, and survival data of adult and juvenile mule deer collected over a 10-year period in western Wyoming, USA, we assessed how attributes of summer range, migratory route, and physical traits of a mother carried over to influence survival of juvenile deer during their first migration and their first winter. We evaluated the hypothesis that carryover effects of a mother’s condition (i.e., age and nutritional condition) and the decisions she makes (i.e., the habitat characteristics on a mother’s summer range and migratory route and the choices she makes while migrating) would influence survival of juvenile deer during migration and winter.

## Materials and methods

### Study area

We evaluated the influence of maternal characteristics of summer range and migratory routes on survival of juvenile mule deer in the Wyoming and Salt River mountain ranges (42°25°N, 110°42°W). Mule deer in the Wyoming and Salt ranges wintered in low elevation (~ 1,800–2,300 m) ranges between December and April (Dwinnell et al. 2019). Vegetation types on winter range were primarily sagebrush steppe (*Artemisia* spp.) and mixed mountain shrub communities (*Juniperus* spp., *Cercocarpus* spp., *Amelanchier* spp., and *Symphoricarpos* spp.). Each spring between late March and early May animals began their migration to their high elevation summer ranges (~ 2,300–2,700 m). Vegetation types on summer ranges ranged from mixed mountain shrub (*Juniperus* spp., *Cercocarpus* spp., *Amelanchier* spp., and *Symphoricarpos* spp.) and sagebrush steppe (*Artemisia* spp.) communities at low elevations to aspen (*Populus tremuloides*), conifers (*Pinus* spp., *Pseudotsuga* spp., and *Abies* spp.), and tall forb (*Geranium* spp., *Lupinus* spp., *Delphinium* spp.) communities at high elevations. Once they have arrived on summer range, deer typically give birth in the first two weeks of June. Animals remained for 5–6 months on summer ranges, before beginning their autumn migration between October and December.

### Animal capture and handling

Beginning in spring 2015, we captured 70 adult, female mule deer using helicopter net gunning (LaSharr et al. 2023b). Following the initial capture event, we recaptured all surviving animals each autumn (early December) and spring (mid-March) until December 2025, and captured new animals to maintain our sample size of 70 adult, female mule deer. At each capture event we fit all animals with GPS radio collars, measured body mass, and estimated nutritional condition through rump fat measurements and body condition scores (Cook et al. 2007; Stephenson et al. 2002). We used developed protocols for mule deer to estimate ingesta-free body fat (hereafter, body fat) of all animals (Cook et al. 2007). Body fat is an important indicator of nutritional condition and can directly influence survival over winter (LaSharr et al. 2023a) and offspring success in mule deer (Lamb et al. 2023). Each spring, we used ultrasonography to evaluate pregnancy and fetal rates, and fit all pregnant animals with vaginal implant transmitters (Aikens et al. 2021). At the initial capture event for each animal, we assigned a unique animal identifier (e.g., “001”) and extracted one incisiform canine to assess age using cementum annuli (LaSharr et al. 2023b).

In late spring of each year (beginning in late May), we monitored pregnant females using both movement data and VIT expulsion alerts to identify birth events. After a suspected birth event occurred, we relocated adult females on their summer range and visually observed them to confirm if the birth event had occurred. Upon confirming a birth event, we searched the surrounding area and captured newborn fawns. Upon capture, we restrained the newborn animal and collected data on mass (kg), sex, and morphometrics (Monteith et al. 2014). We placed an expandable collar on the animal (ATS VHF 2015; ATS neolink 2016–2017; Vectronic Aerospace GPS 2018–2024). We assigned a unique identifier for each newborn deer (e.g., “F001”).

### Summer range

We estimated the proportion of different habitat types during the growing season for adult, female deer. First, we identified the area of a female’s summer range using 95% contours from Brownian Bridge movement models using GPS locations from deer between 1 June and 30 August of each year (Sawyer et al. 2009). We used remotely sensed data of habitat type from LANDFIRE 2016 to estimate the proportion of different habitat types on each summer range (LANDFIRE, 2016). We reclassified vegetation types from LANDFIRE 2016 into functional groups that were relevant to mule deer (Appendix 1) using input and expertise from habitat biologists and land managers with experience in our study area. Tall forb communities are considered high-quality habitat for mule deer in our study area; thus, we calculated the proportion of the tall forbland on summer range of female deer. We calculated cumulative precipitation in the summer from 1 May to 30 August on each home range by summing the daily estimates from DAYMET (a gridded climate dataset providing daily surface weather data at 1 km spatial resolution) for each cell and calculated the average for each home range (Thornton et al. 1997). We used this extended time window beyond the time period we used to classify seasonal ranges because of the influence of early spring precipitation (May - June) on plant growth.

### Quantifying migration metrics

We identified the initiation and end of migration using net-squared displacement for each animal during each season (Bunnefeld et al. 2011; Merkle et al. 2022). During each migratory season, we calculated the average initiation date of migration of all adult animals. We then calculated deviation from the average initiation date by subtracting the initiation date of each individual from the average initiation date of the population. Deviation from mean start date was highly correlated to the Julian date when animals began migrating (*r* = 0.97); we opted to use deviation from mean start date to account for inter-annual variation in environmental conditions that may influence the actual timing of when animals migrate. We defined each migratory route using a line-buffer approach (Merkle et al. 2023) using all steps that occurred during migration not on stopovers (excluded steps > 500 m/h). We then created a 1,000-meter buffer around the migration path (Jakopak et al. 2025). Finally, for each animal, we also calculated the average length of each migration route, the average number of days spent migrating, and the average speed of all migrations preceding the current migration. Using the buffered polygon of each route, we extracted the proportion of agriculture and development (i.e., roads and infrastructure) from the reclassified LANDFIRE categories (Appendix 1). We selected these two categories because they potentially posed higher risk to mule deer while migrating.

### Winter severity

During the study period, animals in this population experienced winters that ranged from mild to severe given the 30-year population average for winter severity (LaSharr et al. 2023a). Once on winter range, juveniles can separate from and sometimes do not spend the winter in the same area as their mothers (Jakopak et al. 2025; unpublished data Wyoming Range Mule Deer Project). We did not have GPS data for all juveniles during all winters, thus we were unable to calculate individual metrics of winter severity for juveniles. Therefore, we classified winters using a binary value of ‘normal’ or ‘severe’ based on observations in the field and extent of adult mortality observed which exceeded 30% of adult, female mule deer during the bad winters (LaSharr et al. 2023a).

### Statistical analyses

We evaluated two a priori hypothesis groups for each season to understand how maternal condition and behavior influenced survival of their juvenile offspring during both the migration season and while on winter range using logistic regression. We used logistic regression instead of a time-to-event survival analysis because timing of migration and arrival to winter range varied among individuals and our primary interest was in understanding the survival outcome during those ecological windows (migration and survival while on winter range) and not necessarily the time to mortality during those windows. Additionally, the timing of exposure was defined by the biological event (e.g., autumn migration), which differed in timing, length, and distance across individuals. During each season, we classified survival of a juvenile as 1 and mortality as 0. We report 90% confidence intervals to reduce the probability of Type II error given the small number of observed juvenile mortalities. All analyses were performed using the glm() function in R version 4.5.3 (R Core Team 2026).

To evaluate the influence of habitat, maternal condition, and maternal behavior on survival during migration, we fit five separate logistic regression models because of the limited sample size of juvenile mortality that occurred during migration (*n* = 23). The migration season was classified from the day that a mother initiated migration and ended the 7 days after their mother ended migration in the autumn. We included the buffer at the end of migration to account for delayed mortality that may have occurred as animals were exposed to novel conditions in new environments. Additionally, we only included juveniles in these analyses whose mothers were alive and survived the entirety of the autumn migration. We excluded juveniles that died within 5 days of capture (if capture occurred during migration) to reduce the possible influence of capture-related mortality, juveniles whose mothers died before completing their autumn migration (e.g., orphans), and juveniles with unknown fates because of collar failure. We included days migrating as a covariate in all five models to account for variation in time at risk across individuals. We evaluated the following five models: (1; route habitat) proportion of agricultural land and developed land on the mother’s migration route; (2; maternal internal state) maternal age and December body fat; (3; litter characteristics) sex of juvenile and if the juvenile had a sibling that survived to 90 days (binary 0/1 for no/yes), (4; maternal migration behavior) deviation from the population mean migration departure date and total migration route length; and (5; null model) including only time at risk. We used December body fat of mothers as a proxy for condition while migrating because migration timing and collection of December body fat typically occurred within ~ 1 month of each other. We did not include random effects because repeated measures per mother were insufficient to support a random intercept or slope (Bolker et al. 2009). Additionally, we evaluated the potential for nonlinear effects of predictors on survival during migration; including quadratic terms did not improve the fit or change the effect size, thus, we did not include them in our final models (Appendix 2).

The winter season was classified as 7 days after their mother ended their autumn migration to 1 May of the following year. We used juvenile mortality during winter as the response variable, and included age of the mother, body fat of the mother in spring prior to when juveniles were born, mass of the juvenile at birth, the proportion of tall forb community on a mother’s summer range, precipitation on summer range, deviation in mean start date of migration from the population average, sex of the juvenile, if the juvenile had a sibling that survived to 90 days (binary 0/1 for no/yes), and winter condition (binary 0/1 for normal/bad) as predictors. Finally, deviation in mean start date of migration from the population average was correlated with number of days on winter range (*r* = 0.47), as mothers that departed later had shorter exposure windows during winter. To evaluate whether the relationship between departure timing and juvenile winter survival reflected a biological carryover effect or simply reduced time at risk, we examined the timing of juvenile mortality during winter. The majority of juvenile mortalities occurred after 31 January (69.4%, *n* = 84 of 121 deaths during winter), and 93.5% of animals (*n* = 186 of 199 juveniles) arrived on winter range before 1 January. We opted to retain deviation in mean departure date in the model and excluded number of days on winter range. We did not include random effects in the model because repeated measures were not sufficient to include a random intercept or slope (Bolker et al. 2009). Additionally, we evaluated the potential for nonlinear effects of predictors on survival on winter range; including quadratic terms did not improve the fit or change the effect size, thus, we did not include them in our final models (Appendix 2).

## Results

We evaluated how maternal attributes, characteristics of summer range, and migratory route of 119 mothers influenced survival of 207 juveniles during autumn migration and how maternal attributes, characteristics of summer range, and migratory behavior of 118 mothers influenced survival of 199 juveniles over 10 summer and winter seasons (2015–2025). Mothers had an average of 6.4% proportion of tall forb communities on their summer range (range 0–35%; median 4.5%) and were exposed to an average of 242.18 mm of precipitation during summer (range 165–309 mm; median 242.54 mm). Mothers migrated for an average of 26 days (range 2–107 days; median 19 days) and an average of 90.5 km (range 13.2–241.5 km; median 87.4 km), and initiated migration on average on 30 October (range 9 August to 10 January). Average age of mothers at birth was 7 years (range 2–14 years; median 7 years) and average body fat in spring was 5.33% (range 0.22% to 13.14%; median 5.50%). Juveniles were on average 3.44 kg at birth (range 2.15 to 4.75 kg). Mothers had an average of 2.5% agriculture (range 0–14.8%; median 1.8%) and an average of 0.6% development (range 0–2.7%; median 0.6%) on their migration routes. Of the 199 juveniles entering their first winter, 112 (56.3%) were female and 87 (43.7%) were male, and 47.7% had a sibling that survived beyond 90 days since birth.

### Maternal habitat choice drives juvenile survival during migration

**Fig. 1:**
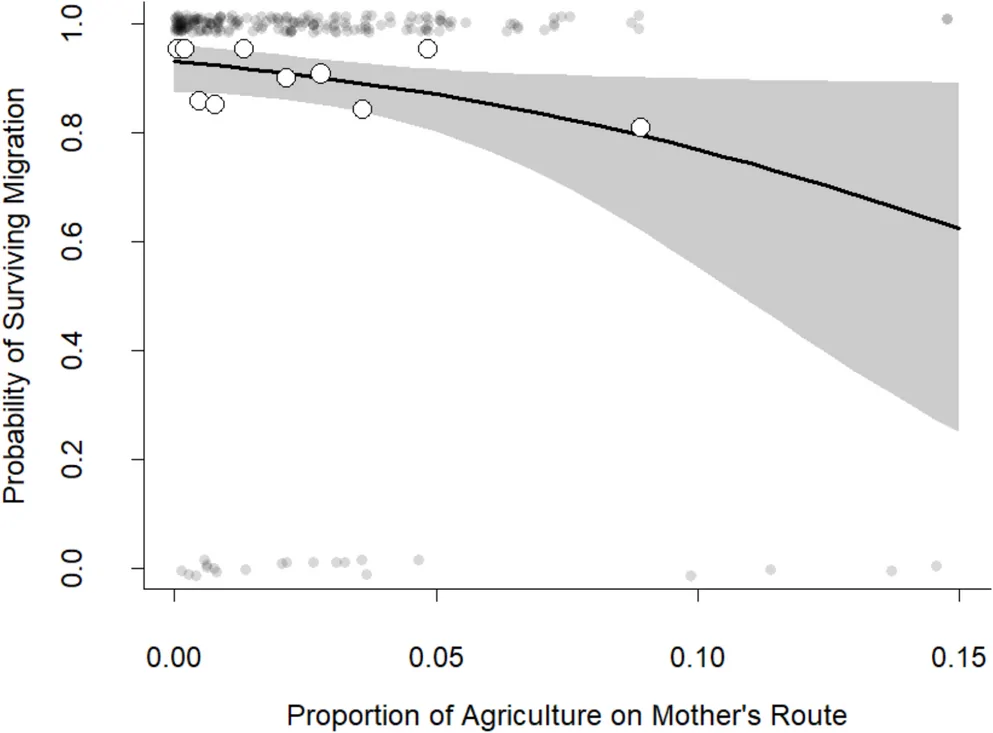
The proportion of agriculture on the migration route of a mother influenced the survival of her 6-month-old juvenile during their first autumn migration event in mule deer (*Odocoileus hemionus*). Model predicted probability of survival and 90% confidence intervals from proportion of agriculture on migration routes. Small dark points represent the jittered, raw data, and large white circles represent the mean observed survival within quantile-based bins of equal sample size. The circle size is proportional to the number of individuals in each bin. Data were collected in western Wyoming, USA from autumn 2015 through autumn 2024

The only characteristics of a mother’s route that affected juvenile survival during migration was proportion of agriculture on the migratory route—animals with migratory routes containing high amounts of agriculture were more likely to have juveniles that died during migration (Table 1; Fig. 1). An increase in the proportion of agriculture from 1% to 10% on a mother’s migratory route increased the probability of mortality of her offspring while enroute from 7.9% to 23.1% (Table 1; β = 13.98, SE = 6.76, 90% CI: 2.40–25.04, *p* = 0.039). Length of route, deviation from the average initiation date, proportion of development on route, and days spent migrating did not influence probability of mortality of juveniles during migration. Further, survival of juvenile deer during migration was not affected by age or condition of a mother in the autumn during migration.

**Table 1:** Estimate, standard error, z-value, p-value, and 90% confidence intervals from a logistic regression model evaluating if a mother’s juvenile survived during migration and winter with data from adult, female mule deer in western Wyoming, USA from autumn 2015 to spring 2025

Season	Model	Predictor	Estimate	SE	z	*P* - value	90% LCI	90% UCI
Migration	Null	Intercept	-2.320	0.367	-6.322	< 0.001	-2.953	-1.741
		Days Migrating	0.005	0.010	0.502	0.616	-0.013	0.021
	Route habitat	Intercept	-2.633	0.495	-5.316	< 0.001	-3.492	-1.855
		Proportion of Agriculture on Route	13.978	6.764	2.067	0.039	2.403	25.041
		Proportion of Development on Route	4.275	47.779	0.089	0.929	-82.123	76.933
		Days Migrating	0.000	0.011	0.019	0.985	-0.019	0.018
	Litter characteristics	Intercept	-2.329	0.496	-4.701	< 0.001	-3.192	-1.554
		Sex of Juvenile (Male)	0.221	0.462	0.477	0.633	-0.550	0.984
		Sibling Survived 90 days	-0.163	0.470	-0.347	0.729	-0.954	0.607
		Days Migrating	0.005	0.010	0.445	0.656	-0.013	0.021
	Maternal state	Intercept	-1.390	1.209	-1.150	0.250	-3.396	0.595
		Mother’s Age (years)	-0.042	0.110	-0.380	0.704	-0.229	0.136
		Mother’s Autumn Body Fat (%)	-0.072	0.077	-0.938	0.348	-0.204	0.050
		Days Migrating	0.005	0.011	0.500	0.617	-0.013	0.022
	Migration behavior	Intercept	-2.454	0.564	-4.349	< 0.001	-3.412	-1.547
		Deviation from Mean Start Date (days)	-0.004	0.013	-0.320	0.749	-0.026	0.017
		Total Migration Distance (km)	0.004	0.008	0.499	0.618	-0.009	0.016
		Days Migrating	-0.003	0.017	-0.174	0.862	-0.033	0.024
Winter		Intercept	4.379	2.099	2.086	0.037	0.981	7.908
		Winter Condition (Bad)	-2.792	0.653	-4.276	< 0.001	-4.014	-1.822
		Mother’s Age (years)	0.217	0.081	2.659	0.008	0.086	0.354
		Mothers Spring Body Fat (%)	0.009	0.085	0.106	0.915	-0.132	0.150
		Precipitation on Summer Range (mm)	-0.006	0.005	-1.104	0.270	-0.014	0.003
		Fawn Mass at Birth (kg)	-0.608	0.345	-1.763	0.078	-1.185	-0.046
		Proportion of Tall Forbs on Summer Range	1.844	3.114	0.592	0.554	-3.184	7.206
		Deviation from Mean Start Date (days)	-0.017	0.008	-2.085	0.037	-0.030	-0.004
		Sibling Survived 90 days	0.719	0.342	2.102	0.036	0.162	1.290
		Sex of Juvenile (Male)	-0.178	0.345	-0.517	0.606	-0.747	0.389

### Maternal carryover drives juvenile survival during winter

**Fig. 2:**
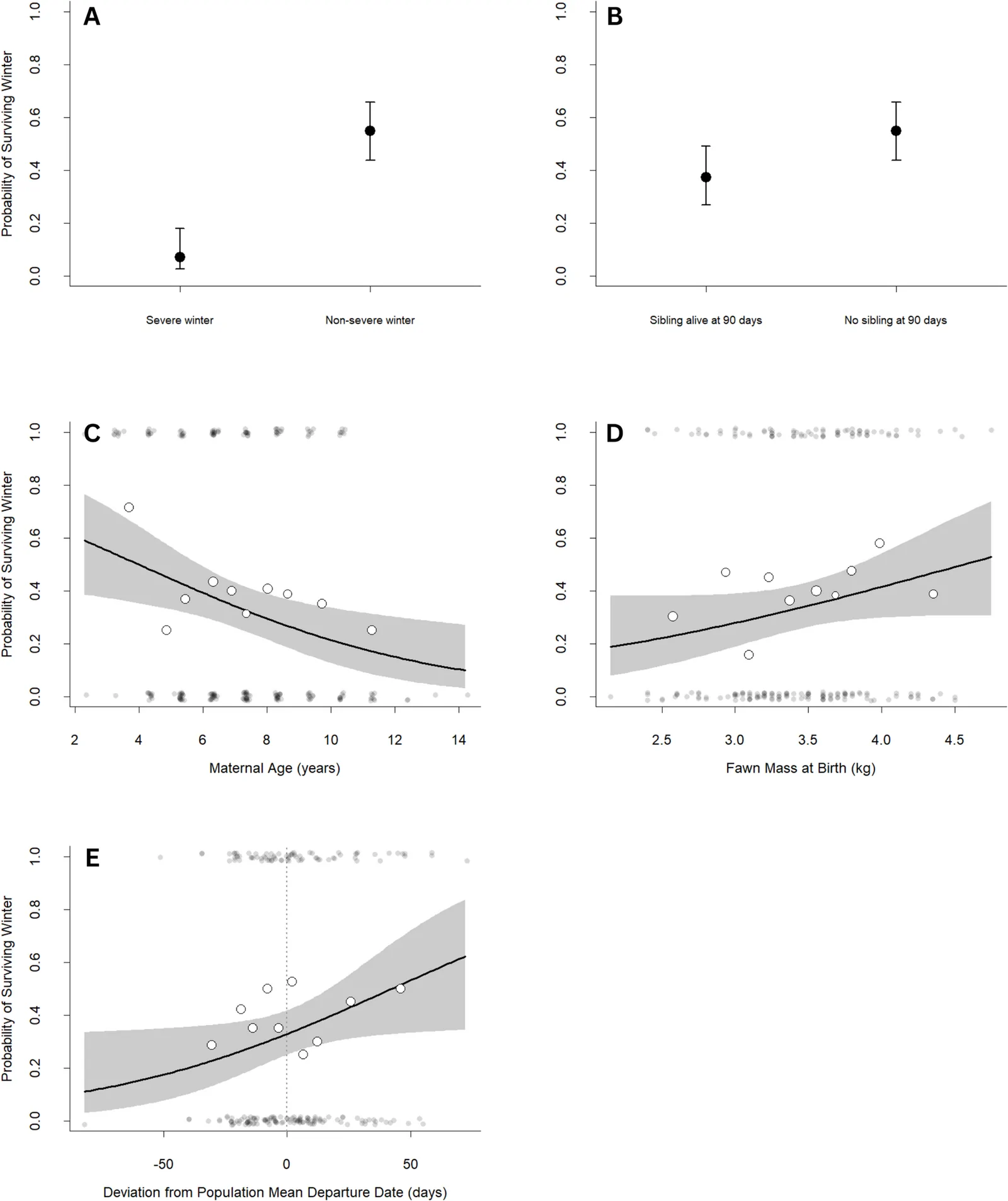
The characteristics and conditions of a mother influenced the survival of her juvenile during their first winter in mule deer (*Odocoileus hemionu*s). Model predicted probability of survival and 90% confidence intervals from severe winters (**A**), sibling status (**B**), maternal age (**C**), mass of neonate at birth (**D**), and deviation in migration departure date from the population average (E). For each predicted probability of survival, all other covariates were held at the median, with no sibling alive at 90 days and a non-severe winter. Small dark points represent the jittered, raw data, and large white circles represent the mean observed survival within quantile-based bins of equal sample size. The circle size is proportional to the number of individuals in each bin. Data were collected in western Wyoming, USA from autumn 2015 through spring 2025

Winter severity had a large effect on overwinter survival (Table 1; Fig 2A; β = −2.79, SE = 0.65, 90% CI: −4.01 to − 1.82, *p* < 0.001), with only 3 of 45 juveniles surviving severe winters compared to 75 of 154 during mild or average winters. Mass at birth, maternal age, deviation from mean start date, and survival of a sibling over summer all influenced survival of juveniles during their first winter (Table 1; Fig. 2). A mother that departed 1 week before the population average decreased her offspring’s probability of over winter survival from 58.6% to 55.7%, whereas a mother who did not depart her summer range until a month after the population average gave her offspring a 70.1% probability of surviving the winter (Table 1; β = −0.017, SE = 0.008, 90% CI: −0.030 to − 0.004, *p* = 0.020). Mass at birth also predicted winter survival, with heavy neonates having a lower probability of mortality (Table 1; β = −0.019, SE = 0.008, 90% CI: −0.032 to − 0.006, *p* = 0.037) than those born small. Maternal age had a positive effect on survival, with older mothers having a higher probability of their juvenile’s dying over winter than young mothers (Table 1; β = 0.217, SE = 0.081, 90% CI: 0.086 to 0.354, *p* = 0.008). Finally, juveniles who had a sibling that survived to at least 90 days had a higher probability of mortality over winter (Table 1; β = 0.719, SE = 0.342, 90% CI: 0.162 to 1.290, *p* = 0.036). Precipitation on summer range, proportion of tall forbs on a mother’s summer range, and maternal condition in spring did not influence overwinter survival (Fig. 2).

## Discussion

The survival of animals from the time they reach independence to their first breeding event plays a critical role in population persistence (Coulson et al. 2001; Gaillard et al. 2000), and though critical, can be an overlooked window of life history when considering parental fitness. For long-lived animals with extended parental care, the choices, experiences, and condition of parents can ultimately determine life or death for young animals. Though typically less obvious, the internal characteristics, habitat, and decisions of mothers carried over and influenced survival of juveniles during their first autumn migration and their first winter. Parents translate their traits, characteristics, and decisions to their young—the route she took between summer and winter ranges became a functional trait of her offspring during their first period of independence, supporting our hypothesis that maternal behaviors carry over to affect juvenile survival in subsequent seasons. Our results demonstrate the importance of considering how carryover effects shape post-weaning juvenile survival when evaluating questions of population dynamics.

Abundant evidence has highlighted the critical, and beneficial role that migratory corridors and habitat play on individual, population, and species persistence (Aikens et al. 2021; Alerstam and Bäckman 2018; Monteith et al. 2018)—carryover effects from conditions or experiences that occur during migration can have far-reaching consequences. Moving rapidly through complex landscapes can incur risk (Sabal et al. 2021) and mothers that migrated along areas with a high proportion of agriculture exposed their offspring to increased risk of mortality during migration. Agriculture has been shown to provide potential benefits to wild animals through buffered or supplemental nutrition (Anderson et al. 2012), but it is possible the altered landscape of agriculture also increases risk to wild animals, particularly those that are young and naïve. Conversion from natural habitat to agriculture may alter landscape through increased fencing or reduced cover, both could increase risk of predation or accidents and may be difficult for young animals to navigate successfully. Indeed, fencing can impede escape of predators by ungulates (Davies-Mostert et al. 2013) and anthropogenic infrastructure alters migratory behaviors of mule deer and affects their ability to track nutritional resources (Aikens et al. 2022; Lendrum et al. 2013; Monteith et al. 2018; Wyckoff et al. 2018). Although proportion of development along migratory routes had no effect on juvenile survival during migration, the proportion of development on migratory routes was low for many animals, and did not exceed 2.7%. Additionally, hunting can influence behavior of animals and could compound the effects of fences in agricultural areas, however, migration in our study largely occurred after the hunting season has ended, and harvest of females and juveniles is largely prohibited. Identifying habitat that hinders survival of juveniles during migration may be an important, but missing, piece of understanding factors limiting populations, particularly if adults are able to navigate those habitats successfully. Mule deer are faithful to the routes they take each year (Sawyer et al. 2019b; Laforge et al. 2025), and mothers that migrate through habitats that are unfavorable for their offspring may incur repeated costs for their entire lives. Yet, even if juveniles do survive their first migration event, the choices a mother made during that migration event (e.g., migration timing) determined if her offspring survived their first winter.

Migration allows animals to exploit high-quality resources and reduce environmental costs throughout the year (Alerstam and Bäckman 2018). Animals should balance the risks and rewards of seasonal ranges by timing their migration to maximize the benefits and reduce the risks associated with different seasons (Martin et al. 2018). Failure to time migration effectively may limit access to high-quality forage or risk exposure to harsh environments, both of which can harbor fitness consequences. Indeed, mothers who initiated migration early in the autumn exposed their juvenile deer to increased risk of mortality the following winter. We suspect mothers that departed their summer ranges early may have reduced the opportunity their offspring had to eat the abundant and high-quality forage on summer range which can be critical to building capital in the summer. Indeed, previous work has demonstrated that the morphology of juvenile animals can be critical to their persistence through winter (Jones et al. 2017; Lamb et al. 2023), and prolonged exposure to summer habitat may be especially important for young animals who are growing rapidly during their first few months of life. Indeed, juvenile mule deer have grown from ~ 3 kg at birth to ~ 50 kg by the time they reach winter range for the first time between 5 and 7 months old (Anderson and Scharf 2014; Heffelfinger and Heffelfinger 2023; Larivée et al. 2010; Lamb et al. 2023). Departing summer range protects animals from being trapped at high elevations by snow, but leaving early can come at the cost of foraging on summer range in places that have greater diversity, abundance and quality of forage compared with winter range (Monteith et al. 2011). For adult animals who have already reached asymptotic body size, departure timing may not be a critical piece of their winter survival if they already acquired sufficient capital. For young animals, prolonged exposure to summer habitat may be critical as they undergo rapid body growth; consequently, leaving early may have an unintended fitness consequence for mothers if it comes at the cost of survival for her offspring in the coming winter.

Despite the importance of summer forage, habitat, and precipitation on gaining capital (LaSharr et al. 2023c), habitat type on a summer range had no influence on juvenile survival during their first winter. Habitat selection of parents can play an important role on survival for dependent young; for example, increased cover can allow young animals to remain undetected to predators when they are most vulnerable to predation (Van Moorter et al. 2009) and abundant forage can buffer the nutritional condition of mothers and offspring during peak lactation (Parker et al. 2009). Yet, the habitat types that mothers inhabited during summer had no effect on survival of her offspring through their first winter. We acknowledge that we evaluated habitat types at a relatively coarse spatial scale; mule deer select for forage at a much finer scale than we were able to measure, and it is possible that plants and fine-scale habitat measures do influence overwinter survival of juvenile deer. Furthermore, regardless of the habitat type a mother occupied on summer range, the mass of her young at birth and the survival of a sibling to 3 months of age had an important influence on overwinter survival of juvenile deer. Mothers in better condition during gestation produce heavier fawns (Lamb et al. 2023; LaSharr et al. 2025), have increased litter size (LaSharr et al. 2025), and have a higher probability of their offspring surviving summer (Lamb et al. 2023) than mothers in poor condition. Thus, even though spring fat of a mother had no direct influence on overwinter survival of juveniles in our study, fawn mass and litter size likely was the mediating effect of that relationship.

Surviving winter in temperate environments can be difficult; animals need both sufficient stores of body reserves and access to forage to survive (Bright Ross et al. 2021; LaSharr et al. 2023a). For young animals that spend the first period of their life growing rapidly, conditions at birth can set the trajectory for both the current season and the rest of their life. Indeed, the condition of a mother during gestation influences the weight of her newborn (Lamb et al. 2023; LaSharr et al. 2025), which can subsequently influence its survival during summer or can influence phenotype for the remainder of its lives (LaSharr et al. 2023d; Michel et al. 2016; Monteith et al. 2009). When resources become limited and snow becomes deep, winter severity can compound the costs of moving through the landscape and accessing food, can exacerbate energy expenditure, and increase the risk of mortality for wild animals (Parker et al. 1984, 2009) and winter conditions can carry over to influence recruitment through maternal condition in subsequent years (Segar and Keane 2020, Schooler et al. 2022). Navigating harsh winters can be difficult for adults, but may be near impossible for juveniles that lack sufficient somatic reserves to buffer against extreme temperatures and have shorter legs that make navigating deep snow energetically costly. In harsh winters, the probability of juveniles surviving was exceptionally low regardless of the condition of a mother before parturition—only 6.6% of juveniles that were exposed to harsh winter conditions in their first year of life survived the winter season. Winter severity may have extended, multi-year consequences for maternal fitness. Beyond the immediate consequences of harsh winters on juvenile survival, severe winter conditions can limit the nutritional condition of mothers during gestation (LaSharr et al. 2023c) which carries over to influence survival of the offspring she is gestating—consequently, mothers may lose multiple cohorts of offspring when exposed to harsh winters.

How conditions, behaviors, and decisions in one season influence fitness later in life is an important aspect of understanding population dynamics (O’Connor and Cooke 2015; Hayes et al. 2026). Reproduction and survival are the two primary mechanisms that regulate populations (Gaillard et al. 2000; Stearns 1992) and understanding the mechanisms that influence both survival and reproduction is the cornerstone of wildlife management and conservation. Nutritionally dependent young (i.e., pre-weaning) and adult animals are two demographics often considered when determining the factors that regulate populations. Yet, juvenile animals that have reached nutritional independence (i.e., post-weaning) still carry the costs and face the consequences of their mother’s decisions and should be equally considered when evaluating population dynamics and growth. The decisions and conditions of a parent become a functional trait of their offspring even after young animals have reached independence. Carryover effects from mothers to independent juveniles may be a missing link in understanding questions of fitness and basic inquiries into the factors regulating changes in a population.

## Supplementary Information

Below is the link to the electronic supplementary material.

Supplementary Material 1

Supplementary Material 2

## Data Availability

The data used during the current study are available from the corresponding author on reasonable request.

## Code availability

The code used during the current study are available from the corresponding author on reasonable request.
