# Supplementary material for: Maternal carryover drives post-weaning survival of juveniles in a long-lived mammal
Source: Oecologia. 2026 Aug 7;208(9):109. doi: 10.1007/s00442-026-05953-y (PMC13451305; doi:10.1007/s00442-026-05953-y)
Supplement: Supplementary file 1 [file 442_2026_5953_MOESM2_ESM.docx]

Appendix 2.

To evaluate the potential for nonlinear effects of our continuous predictors on juvenile survival, we added a quadratic term for each continuous predictor to its respective candidate model and compared model fit using AICc and evaluated if fit had a biologically meaningful change to effect size. We retained a quadratic term only if it improved AICc by more than 2 units relative to the linear model and the quadratic term itself was statistically or biologically meaningful.

No quadratic term improved model fits more than 2 AICc units relative to the linear version of the same model (Table S1), thus, we retained linear relationships for all continuous predictors in our final models.

Table S1.

| Season | Model | K | AICc | delta_AICc |
| --- | --- | --- | --- | --- |
| Migration | Null | 2 | 139.71 | 0 |
|  | Route habitat | 4 | 139.96 | 0 |
|  | Route habitat + I(proportion of agriculture²) | 5 | 140.31 | 0.35 |
|  | Route habitat + I(proportion of development²) | 5 | 138.88 | -1.08 |
|  | Maternal state | 4 | 142.91 | 0 |
|  | Maternal state + I(mother's age²) | 5 | 144.9 | 1.99 |
|  | Maternal state + I(mother's autumn body fat²) | 5 | 142.59 | -0.32 |
|  | Migration behavior | 4 | 143.46 | 0 |
|  | Migration behavior + I(deviation from mean start date²) | 5 | 145.21 | 1.75 |
|  | Migration behavior + I(total migration distance²) | 5 | 145.49 | 2.03 |
|  | Litter (sex + sibling) | 4 | 143.49 | 0 |
| Winter | Full model | 10 | 235.21 | 0 |
|  | Full model + I(mother's age²) | 11 | 237.3 | 2.09 |
|  | Full model + I(mothers spring body fat²) | 11 | 236.52 | 1.31 |
|  | Full model + I(precipitation on summer range²) | 11 | 236.67 | 1.46 |
|  | Full model + I(fawn mass at birth²) | 11 | 237.34 | 2.13 |
|  | Full model + I(deviation from mean start date²) | 11 | 237.45 | 2.24 |
